# 

**Published:** 1890-03

**Authors:** 


THE FORTY-FIRST ANNUAL MEETING of the American Medical Association will be held in the City of Nashville, commencing Tuesday, May 20th, and continuing until Friday, the 23d. In connection with the meeting of the Association, there will be held the usual exposition of pharmaceutic, surgical, and sanitary products and appliances. This exposition is expected to be one of the largest and most interesting exhibits of the kind ever held. Pharmacists and others intending to exhibit their manufactures, etc., should address Dr. J. Berrien Lindsley, Chairman of the Sub-Committee on Exhibits, Nashville, Tenn. The building selected for the exhibition is Amusement Hall, Broad street, near Spruce, and contains 7,000 square feet of floor space, exclusive of the aisles, and about 5,000 square feet of wall space.—*Memphis Medical Monthly.*

### BOOKS AND PAMPHLETS RECEIVED.

Spinal Concussion: Surgically Considered as a Cause of Spinal Injury, and Neurologically Restricted to a Certain Symptom Group, for which is Suggested the Designation Erichsen's Disease, as one of the Traumatic Neurosis. By S. V. Clevenger, M. D., Consulting Physician to the Reese and Alexian Hospitals, Chicago, etc. Philadelphia and London: F. A. Dovis. 1889. Cloth, 8vo. pp. iv., 359. Price, $2.50, net.

Animal Alkaloids. The Ptomaines, Leucomaines, and Extractives in their Pathological Relations. By Sir William Aitken. Bart., M. D., LL. D., F. R. S. Second edition. Philadelphia: P. Blakiston, Son & Co. 1889.

A Manual of Organic Materia Medica. Being a guide to Materia Medica of the vegetable and animal kingdom, for the use of Students, Druggists, Pharmacists, and Physicians. By John M. Maisch, Ph. M., Phar. D. Fourth edition. With 259 illustrations. Philadelphia: Lea Brothers & Co. 1890.

Hand-book of Materia Medica, Pharmacy, and Therapeutics, including the Physiological Action of Drugs, the Special Therapeutics of Disease, Official and Extemporaneous Pharmacy, and Minute Directions for Prescription Writing. By Samuel O. L. Potter, M. A., M. D. Second edition. Revised and enlarged. Philadelphia: P. Blakiston, Son & Co. 1890.

Transactions of the American Association of Obstetricians and Gynecologists, for the Year 1889. Philadelphia: William J. Dornan, Printer.

Diseases of Women and Abdominal Surgery. By Lawson Tait, F. R. C. S. Edin. and Eng., L. L. D. Vol. I. Philadelphia: Lea Brothers & Co. 1889.

The Medical Register of New York, New Jersey, and Connecticut, for the year commencing June 1, 1889. Published under the supervision of the New York Medico-Historical Society. William S. White, M. D., Editor. Vol. XXVI. New York: G. P. Putnam's Sons. 1888.

Translations of the Medical Association of the State of Missouri, at its Thirty-second Annual Session, held at Springfield, Mo., May 21, 1889.

The Principles and Practice of Surgery. Being a Treatise on Surgical Diseases and Injuries. By D. Hayes Agnew, M. D., L. L. D. Profusely illustrated. Second edition, thoroughly revised, with additions. In three volumes. Volumes I., II., and III. Philadelphia: J. B. Lippincott Company. 1889.

Syllabus of the Obstetrical Lectures in the Medical Department of the University of Pennsylvania. By Richard C. Morris, A. M., M. D., Demonstrator of Obstetrics in the University. Philadelphia: W. B. Sanders. 1890. Price, $2.00, net.

First Annual Report of the State Commission in Lunacy, for the State of New York. Albany: James B. Lyon, State Printer. 1890. Through Hon. Leroy Andrus.

Ninteenth Annual Report of the Managers of the Buffalo State Asylum for the Insane, for the Year 1889. Albany: James B. Lyon, State Printer. 1890.

Thirty-seventh Annual Report of the Young Men's Christian Association, of Buffalo, for the year ending September 30, 1889. Published by the Association. Baker, Jones & Co., Printers. 1889.

Report of Ninety cases of Rapid Dilatation of the Uterine Canal for the Cure of Dysmenorrhea and Sterility. By Franklin Townsend, M. D. Albany, N. Y. Reprint from the Transactions of the American Association of Obstetricians and Gynecologists. 1889.

A Case of Extra-Uterine Pregnancy. Operation. Recovery. By L. S. McMurtry, M. D. Louisville, Ky. Reprint from the Transactions of the American Association of Obstetricians and Gynecologists. 1889.

A Study of the Pathology and Treatment of Intra-Pelvic Inflammations. By L. S. McMurtry, M. D. Louisville, Ky. Reprint from the Transactions of the American Association of Obstetricians and Gynecologists. 1889.

Speech of Mr. Ingalls, of Kansas, in the Senate of the United States, Thursday, January 23, 1890, on the Bill (S. 1121) to provide for the emigration of persons of color from the Southern States.

Our choice of Methods in the Treatment of Uterine Cancer. By Reeves Jackson, A. M., M. D. Professor of Gynecology in the College of Physicians snd Surgeons, of Chicago. Reprint from the *Medical News*, January 18, 1890.

The Application of Forceps to Transverse and Oblique Positions of the Head. Description of a New Forceps. By Henry D. Fry, M. D., of Washington, D. C. Reprint from the *Journal of the American Medical Association*, November 9, 1889.

A Successful Case of Laparatomy and Supra-Vaginal Amputation of the Uterus for Rupture. By Henry C. Coe, M. D., M. R. C. S., Professor of Gynecology at the New York Polyclinic, etc. Reprint from the *Medical Record*, November 2, 1889.

Cornell University, College of Agriculture. Bulletin of the Agricultural Experiment Station. All Departments, xv. December, 1889. Sundry investigations made during the year. Published by the University, Ithaca. 1889.

Rede zur Feier des 75 jährigen Bestehens der Königlichen Frauenklinik zu Dresden gehalten in der Festsitzung der Dresdner Gynäkologischen Gesellschaft, Donnerstag, 5. December 1889. Von Professor Dr. Leopold, Ober-Medicinalrath, Director der Königl. Frauenklinik und Mitglied des Königl. Landes Medicinalcollegiums in Dresden. Dresden: Wilhelm Baensch Verlagshandlung. 1890.

Medical Department, University of Worcester. Announcement for 1890. Cleveland, O., 1889.

State Board of Health Bulletin. Nashville, Tenn., January 20, 1890. Vol. V. No. 6.

Health in Michigan, for January, 1890. Henry B. Baker, Secretary State Board of Health, Lansing.

State Board of Health of New York. Monthly Bulletin for December, 1889, with summary of Mortality for the year 1889.

Summary of Deaths in Newport, R. I., for the Year 1889; compared with those of 1886, 1887, and 1888. By order of the Board of Health.

Weekly Abstracts of Sanitary Reports, U. S. Marine Hospital Service. Vol. V. No. 5, 6, 7, and 8.

---

### Literary and Other Notes.

THE N. Y. PHYSICIANS' MUTUAL AID ASSOCIATION is a benevolent organization for the assistance of sick and destitute members, and the families of deceased associates.

During the twenty-one years of its existence it has paid nearly $60,000 to beneficiaries.

The permanent (benevolent) fund is about $15,000, the income from which may be paid to sick and destitute members.

During the past year the membership has increased from 505 to 730, and the amount payable at death will soon be $1,000.

Expense of membership the past year has averaged less than $1.00 per month.

Any member of the regular medical profession in the State of New York, if under fifty years of age, may become a member.

Initiation fee ($2.00) and first assessment ($1.00) required in advance.

The officers for 1888-90, are: President, Daniel Lewis, 62 Park avenue; First Vice-President, William W. Reese, 175 Hicks street, Brooklyn; Second Vice-President, Charles E. Hackley, 59 West 36th street; Recording Secretary, N. G. McMaster, 322 East 15th street; Assistant Secretary, W. F. Chappell, 22 East 42d street; Corresponding Secretary, Morris H. Henry, 581 Fifth avenue; Treasurer, Robert Campbell, 262 West 23d street. Directors: 1887 90, G. A. Peters, J. L. Vandervoort, A. N. Brockway; 1888-91, M. Blumenthal, G. G. Wheelock, W. F. Cushman; 1889-92, O. B Douglas, D. Magie, W. F. Mittendorf.

We commend this important charity to our readers, and earnestly invite their serious attention to its usefulness.

---

THE modern magazine may be taken as embodying the best literature of the world, as the magazine editor pays the highest prices to novelists, scientists, statesmen, soldiers, and even kings and princes, for the best they can furnish in the literary line. The well-edited magazine becomes an educating influence in the family circle, whose importance cannot be overestimated. The children, as they grow up, are attracted by its illustrations, and so come in time to have a taste for reading. There is always something that is new, something that is strange, something that is interesting; and we consider that we are doing our readers a positive benefit if we are instrumental in placing such a publication within their reach. The special arrangement which we have made with the *Cosmopolitan* presents very unusual inducements. That magazine, although only in the eleventh month under its new management, is already recognized as one of the most interesting publications of the day. It is seeking subscribers everywhere and obtaining them. The proprietors believe that the *Cosmopolitan* has only to be examined to secure a permanent subscriber. That is why we are enabled to make, if the offer is accepted before January next, such a very low rate, by which our readers can obtain the *Cosmopolitan* for little more than the cost of this journal alone. Just think of what the combination means! You obtain your own home journal at about the regular price, and have thrown in a magazine which gives you, in addition, nearly 1,400 pages yearly of reading matter by the ablest writers of the world, including 600 pages of illustrations that are unsurpassed in point of interest and execution. Will it not pay you to send a subscription to this office for the BUFFALO MEDICAL AND SURGICAL JOURNAL, and the *Cosmopolitan*, immediately? Remember, only $3.00 for the two.

